# Date yogurt supplemented with *Lactobacillus rhamnosus* (ATCC 53103) encapsulated in wild sage (*Salvia macrosiphon*) mucilage and sodium alginate by extrusion: The survival and viability against the gastrointestinal condition, cold storage, heat, and salt with low pH

**DOI:** 10.1002/fsn3.4304

**Published:** 2024-08-01

**Authors:** Mahsa Abbasi Saadi, Seyed Saeed Sekhavatizadeh, Hassan Barzegar, Behrooz Alizadeh Behbahani, Mohammad Amin Mehrnia

**Affiliations:** ^1^ Department of Food Science and Technology, Faculty of Animal Science and Food Technology Agricultural Sciences and Natural Resources University of Khuzestan Mollasani Iran; ^2^ Department of Food Science and Technology Fars Agricultural and Natural Resources Research and Education Center, AREEO Shiraz Fars Iran

**Keywords:** extrusion, heat stress, *Lactobacillus rhamnosus*, *Salvia macrosiphon*, simulated gasterointestinal condition

## Abstract

The efficacy of probiotics in providing health benefits may be related to their ability to survive at a sufficient concentration of 10^6^ CFU/g during storage in food and colonization in the gastrointestinal tract. Microencapsulation is a viable method to improve the survivability of probiotics under harsh environmental conditions. In this research, microencapsulated *Lactobacillus rhamnosus* (MLR) was produced by a two‐layer extrusion technique with sodium alginate and wild sage (*Salvia macrosiphon*) mucilage (SMM) in varying concentrations ranging from 0.2% to 0.8% as the first and second wall materials, respectively. The microencapsulation efficiency and second layer diameter of beads increased significantly with the increase in SMM concentrations. Microencapsulated *Lactobacillus rhamnosus* (LR) maintained its minimal concentration (6 log CFU/g) during 9 min at 72°C. The MLR‐date yogurt (DY) sample had the lowest pH, highest acidity, and highest survival rate among the others at the end of storage. In simulated gastrointestinal conditions (SGC), the survival rates of free LR (FLR) and MLR were 45% and 47% on the 14th day of storage, respectively. In sensory properties, MLR had the highest score in odor and texture parameters but not in others. The MLR viscosity (666.3 mPa·s^−1^) and SEM images show a relatively denser structure for MLR. In conclusion, this study emphasized the potential of using double‐layered beads to protect probiotics, providing valuable inspiration for developing new functional foods with higher survival ability in harsh conditions.

## Graphical abstract

MLR maintained its minimal concentration (6 log CFU/g) for 9 min at 72°C. The use of alginate and SMM can protect LR against harsh environmental conditions. MLR‐DY sample had the lowest pH, highest acidity, denser structure, and highest survival rate among the others on the 28th day of storage.

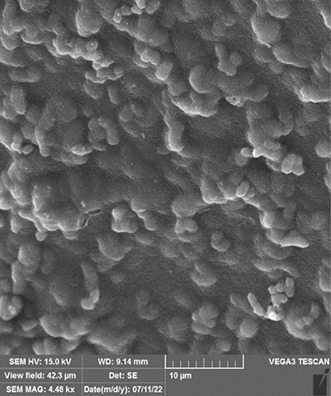

## 1 INTRODUCTION

According to the WHO/FAO definition, probiotics are living microorganisms; when present in sufficient amounts in food, they may reach the intestine and maintain the microbial flora balance. Intestinal health, boosting the immune system, providing anti‐cancer and anti‐diarrheal effects, hypocholesterolemic effects, and reducing lactose in dairy food for lactose intolerance patients are the most important benefits of probiotic bacteria. Probiotic bacteria mainly belong to the *Lactobacillus* and *Bifidobacteria* genera (Hwang et al., 2023). LR is a lactic acid‐producing, non‐spore‐forming, and Gram‐positive bacterium that is categorized as a favorable organism in the gut (Fuad et al., 2023).

This species is suitable for human consumption, and it can survive in food products such as orange juice, but it is unable to grow during storage. In addition, studies have shown that it improves the sensory properties of grape juice (Küçükgöz & Trząskowska, 2022). LR is used in dairy products such as chestnut mousse, puddings, and dairy desserts (Dokoohaki et al., 2019).

Foods that contain these bacteria are classified as functional or beneficial foods. Based on the International Federation of Dairy Products recommendation, these foods must contain a limited probiotic number of 10^6^ CFU/mL (Sangkam et al., 2023). However, they are adversely affected by harsh conditions as well as the commensal microflora of the host, low pH, osmotic pressure, oxidative stress, and bile salts. Thus, probiotics may have difficulty surviving during storage and GI conditions (Kathiriya et al., 2023). Therefore, the bacteria must keep themselves above the necessary limit to reach the intestine and colonize there (Feng et al., 2020). One of the ways to maintain the limited number of probiotics and increase the survival rate in harsh conditions is through microencapsulation. Microencapsulation is a technique that entraps bacteria or active substances in a wall polymer matrix. This technique can protect bacteria and increase the survival rate in storage and GI conditions (Nezamdoost‐Sani et al., 2023). Several techniques can be applied for encapsulation, like coacervation, freeze drying, extrusion, fluid bed, spray chilling, and emulsification (Anjum et al., 2023). In the extrusion method, bacteria are mixed with a sodium alginate solution, which is then added to calcium chloride using a syringe. The extrusion technique has many advantages, for example, high cell survival ability, uniform bead size, mild process conditions, low cost, and simplicity (Huang et al., 2023).

One of the materials used for encapsulation is alginate. Alginate is an ingestible polymer that is employed as a wall material for the entrapment of probiotic cells. It is sensitive to acid, therefore, it cannot survive in the stomach. In this regard, it is suggested to use a second‐layer polymer to promote wall resistance in gastric conditions (Ali et al., 2023).

The different sources of seed mucilage include sage seeds, barhang, wild plum, psyllium, fenugreek, cassia, mesquite, tamarind, balangu, camelina, flaxseeds, chia seeds, quince, cress, and basil (Gao et al., 2024). Mucilages can be used as wall materials in the microencapsulation of probiotic bacteria. These materials can be dissolved in water, degrade slowly, and have the ability to form films. The plant mucilages can be used as wall materials in beads and may be combined with alginate to increase their suitable properties in encapsulation. Moreover, they have a positive effect on the natural colon flora (Cakmak et al., 2023).

Wild sage mucilage has not been used for microencapsulation yet. One of the most important members of the *Lamiaceae* family is wild sage (*Salvia macrosiphon*). *Salvia macrociphon* Boiss is a perennial plant, almost greenish‐white or yellow‐green, slightly fragrant, covered with dense, non‐tuberous fleecy, tuberous, and slightly hairy at the ends (Banan et al., 2023). It belongs to the *Salvia* genus. It is a perennial, herbaceous, and strongly aromatic (lemon‐scented) plant. Multiple pharmacological effects, like inhibitory effects on tumor cell lines, antimicrobial properties, anti‐inflammatory activities, and strong antioxidant activity, are seen in *S. macrosiphon*. (Valifard et al., 2015).

Natural preservatives for fish, poultry, meat, and condiments, as well as the antibacterial properties of wild sage, make it a valuable plant. Recently, wild sage extracts have been used as food shelf‐life enhancers because of their high antioxidant activity. On average, wild sage has 69.01% carbohydrate, 2.08% protein, 9.20% ash, 11.24% moisture, and 30.2% uronic acid. The polysaccharide of the wild sage is a galactomannan with an average molecular weight of ∼4 × 10^5^ Da and a 1.78–1.93:1 mannose/galactose ratio. The existence of carboxyl groups within the framework may serve as locations for the attachment of ions (Razavi et al., 2014).

The oldest tree cultivated is the date palm ( *Phoenix dactylifera* L.). It originates in Arabian countries with more than 7000 years of consumption history. The date palm belongs to the *Arecaceae* or *Palmae* families. The date products (including date paste and date syrup) and date fruit have excellent nutritional value with unique functionality because of digestible sugars such as sucrose (3.2%–7.4%), glucose (42.3%–51.8%), fructose (22.5%–47.5%), dietary fibers (2.2%), proteins (1.8%–30.0%), mineral salts, vitamins, lipids, and pectin. Consequently, different products such as yogurt, chutney, candy, jam, and jelly can be produced from date fruits (Ali et al., 2023).

Date juice is made by dissolving and diluting the soluble solids of date in water and removing the insoluble solids. In this operation, stirring, heating, and macerating the date can increase the yield of the process. This product is sometimes consumed as a drink (Ashraf & Hamidi‐Esfahani, 2011).

The primary objective of the present investigation was to enhance MLR survival following heat stress. Our research has predominantly concentrated on microencapsulation LR with SMM‐alginate used as wall materials in the extrusion technique. The survival of MLR and its physicochemical properties were analyzed. Additionally, MLR and FLR survivability during SGC and DY storage were evaluated. Besides, the sensorial and physicochemical properties of probiotic DY were assessed.

## 2 MATERIALS AND METHODS

### 2.1 Materials

Wild sage (*Salvia macrociphon* Boiss) was harvested in the northeast of Tehran. It was prepared in Ahura Med, Marvdasht, Fars, Iran. *Lactobacillus rhamonosus* (ATCC 53103) was prepared from the Persian Type Culture Collection, Tehran, Iran. Sodium citrate, peptone water, MRS Agar, MRS broth, HCl, rhamnose, NaOH, NaH_2_PO_4_·2H_2_O, and Anaerocult® A Merck Gas Pack were purchased from Merck company (Merck, Darmstadt, Germany). Sodium alginate, pepsin (derived from porcine stomach mucosa), lipase (from *Rhizopus oryzae*), bile (bovine bile), and pancreatin (from porcine pancreas) were prepared by Sigma Company (Steinheim, Germany). Date juice was purchased from Minoo CO., Shiraz, Iran. Low fat milk and skim milk powder were obtained from Pegah Dairy CO. Fars, Iran. Starter culture (CH1‐DVS‐50 U) was purchased from Christian Hansen (Denmark).

### 2.2 Mucilage extraction

SM was purchased from a local producer. The identification of SM was carried out in the herbarium section of the Fars Agricultural and Natural Resources Research and Education Center. The extraction was done by the maceration method. The ratio of water to seeds was 20:1 at pH = 7. 50°C, and 20 min in a stirrer (IKA, RCT basic). The SM was dehydrated in an oven at 50°C. After that, it was milled with an electric miller (Moulinex, France) (Zameni et al., 2015).

### 2.3 LR cultivation

To activate LR, MRS broth medium was used. To create an anaerobic environment, sterile paraffin with a diameter of 5 cm was used. The tube was kept at 37°C for 72 h. The novel selective medium‐modified rhamnose 2,3,5‐triphenyl tetrazolium chloride–LBS–vancomycin agar (M‐RTLV agar) was used for LR. The plates were incubated at 37°C for 48 h under anaerobic conditions using an anaerobic jar and an Anaerocult® A Merk Gas Pack (Dokoohaki et al., 2019).

### 2.4 LR microencapsulation procedures

After activation, the cultured LR in the MRS were centrifuged at 4500 rpm for 10 min; the bacterial sediment was washed twice with sterile 0.1% peptone water. The washed bacterial cells were brought to a 5 mL volume using normal saline. The extrusion technique was used for double‐layer microencapsulation. The sodium alginate and SMM were chosen as the first layer and the second layer wall material, respectively. For this purpose, after centrifugation, 5 mL of the precipitated washed bacterial culture at a concentration of about 3.8× 10^10^ CFU/mL was dissolved in 15 mL of 1.5% sodium alginate solution. Then, it was added drop by drop to 50 mL of sterile CaCl_2_ 0.1 M solution using a 0.11‐mm needle. The produced beads were placed in the refrigerator overnight. In the next step, 1.0% sterile peptone water was applied to wash the beads. Then, the beads were added to 100 mL of SMM mucilage solution in four concentrations (0.2%, 0.4%, 0.6%, and 0.8%), respectively. The solutions were shaken at 30 rpm for 40 min. In the next step, the beads were placed and rinsed with 0.1% sterile peptone water several times.

The size and shape of the beads were obtained from the microscope (Olympus Optical Microscope BX51, Japan) image and microscope measurement version 1.07 software. The aspect ratio was measured using Equation 1 (Pourakbar et al., 2023):

(1) $$\text{Aspect Ratio}=\text{Major axis}\;\left(\mathrm{mm}\right)/\text{Minor axis}\;\left(\mathrm{mm}\right).$$

### 2.5 SEM

The lyophilized samples were fixed on an aluminum holder and sprayed with gold (Desk Sputter Coater DSR1, Nanostructural Coating Co., Iran) before being examined under a scanning electron microscope (SEM, VEGA3, TESCAN, Czech Republic). Then, the samples were observed under a voltage of 0.10 kV. During operation, the distance between the microscope lens and the sample surface was 8.91–7.03 mm (Sekhavatizadeh et al., 2023).

### 2.6 Color

A colorimeter (Konica Minolta CR400, Japan) was used to evaluate the color of the beads. The device was calibrated as standard a* = 23.92, L* = 1.29 and b* = 1.19. For measurement of each color's parameters, 5 g of beads were placed inside a special chamber, and the components of brightness (L*), tendency to yellowness (b*), and tendency to red (a*) were measured for each bead sample.

### 2.7 Heat resistance of FLR and MLR

Free and microencapsulated bacteria were inoculated into the MRS broth medium and placed in a hot water bath at 72°C. Then the samples were selected (0, 3, 6, 9, 12, and 15 min) for dilution and cultured in MRS agar medium under anaerobic conditions (Karimi et al., 2021).

### 2.8 Survival ability of MLR and FLR at 4°C

The FLR and MLR were cultured and maintained in MRS broth at 4°C for 28 days. The LR cell survival ability was assessed on the 1, 7, 14, 21, and 28th days of the storage period.

### 2.9 DY production

For the production of stirred date yogurt, low‐fat pasteurized milk (0.5%) was used. All powdered materials, including starch and skim milk powder, were added to the milk at a rate of 2.5% at 40°C and stirred until the mixture reached 60°C. Then the milk was heated at 90°C for 10 min. Next, the mixture was cooled to 45°C. After cooling, CH1 starter culture, including *Lactobacillus bulgaricus* and *Streptococcus thermophilus* (as a ratio of 1:1), was added to the mixture. Pasteurized date juice (15%) was added to yogurt and stirred for 2 min. The FLR and MLR‐DY were prepared using the same procedure. The final approximately equal numbers of LR cells in MLR and FLR‐DY were about 9.1 log CFU/g. The DY samples were poured into a sterile package. The microbial analysis, sensory, and physicochemical properties of all produced DY were measured during storage at 4°C. The DY samples were kept at 4°C for 28 days. The microbiological, organoleptic, and physicochemical properties, including pH, and titratable acidity, were evaluated on the first day and during storage at 7‐day intervals for 21 days. The survival of LR during the GI transition, rheological, and color parameters were measured on 1 and 21 days of storage time (Ali et al., 2023).

### 2.10 Acidity and pH of DY

A pH meter (Greisinger Electronic, Germany) was used to measure pH. The DY acidity was measured by the titration method (Sahan et al., 2008).

### 2.11 Sensory analysis

For descriptive sensory analysis of the DY samples, 52 panelists were selected. To minimize any sensory distractions, sensory tests were carried out under equal feeding, lighting, and housing conditions. The score table “Hedonic 5‐point” was used for points 1 (strongly opposite) to 5 (strongly agree) (Karimi et al., 2021).

### 2.12 Rheological properties

Rheological studies were carried out on DY samples stored at 4°C using a rheometer (MCR 302, Anton Paar, Austria). Five milliliters of the sample was poured into concentric cylinder geometry at a shear rate of 10–100/s at 23°C.

### 2.13 Starter culture and LR counting

The M‐RTLV was used to count LR. Sterile trisodium citrate (225 mL) (2% w/v) was used to dilute yogurt samples (25 g) at 40°C. *Lactobacillus bulgaricus* and *Streptococcus thermophilus* were cultured in M17 and MRS medium at pH = 5.5, respectively (Borhanpour et al., 2021).

### 2.14 Survival of FLR and MLR exposed to SGC

Dokoohaki et al. (2019) method was applied for assessment of FLR and MLR survival in SGC, which contained simulated gastric fluid (SGF) and intestinal fluid (SIF). FLR and MLR were selected in two quantities of 1 g and 0.1 g for this measurement. The FLR and MLR were added to flasks containing SGF and placed in a shaker incubator (shaker incubator, Labtron, Tehran, Iran), set at a temperature of 37°C for 2 h (SGF phase). Following that, the materials were included in SIF at pH 4.3–5.2. The LR counts were assessed three times after 0.5 h, 2 h, 4 h, and 6 h (each in three separate flasks of the same experiment) using different volumes (0.01 to 1 mL change). Samples of 0.01 mL and 0.1 mL from each solution were immersed in a selective medium. Then, they were incubated under anaerobic conditions at 37°C for 48 h. The survival rate was determined using Equation 2:

(2) $$\text{Survival rate}\;\left(\%\right)=\left(\mathrm{CFU}\;\mathrm{Log}\;N\right)/\left(\mathrm{CFU}\;\text{LogN0}\right)\times 100$$

where *N*, the number of viable LR in CFU/mL after exposure to SGF or SIF and N0: the number of viable LR in CFU/mL before exposure to SGF or SIF (Dokoohaki et al., 2019).

### 2.15 Statistical analysis

The data were analyzed using (SPSS version 22) software. All experiments were performed in triplicate. To statistically analyze the data during the storage period, a one‐way analysis of variance was used, and Duncan's test was applied to check the difference between the means at the level of 5% error probability. Excel 2019 software was applied to draw graphs.

## 3 RESULTS AND DISCUSSION

### 3.1 Encapsulation efficiency, color, morphology, and size of beads

**FIGURE 1:**
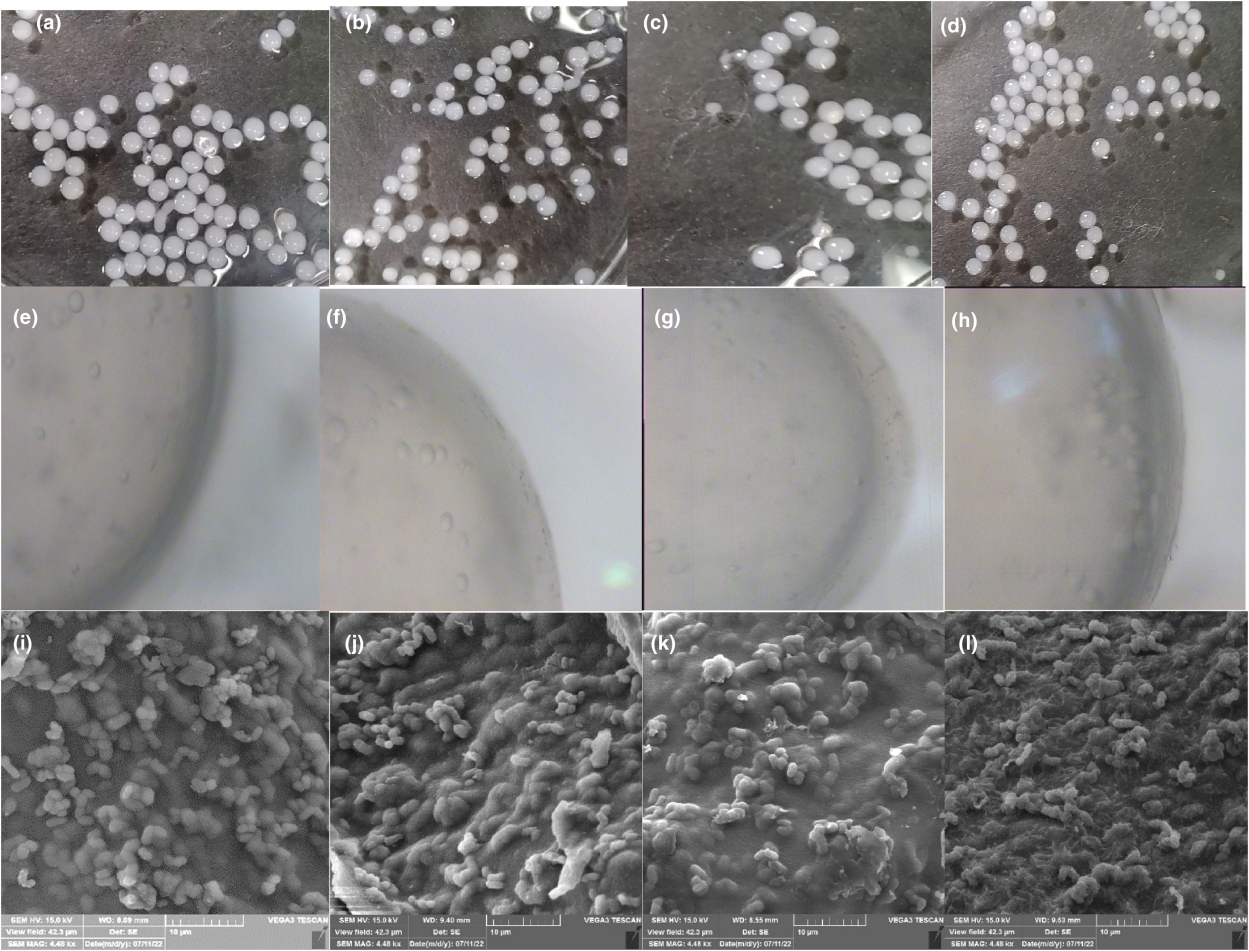
Photography image of microencapsulated *Lactobacillus rhamnosus* (MLR) from left to right, at concentrations of 0.2, 0.4, 0.6, and 0.8 percent (a–d) with *Salvia macrosiphon* mucilage (SMM), respectively (top row); and light microscopy of MLR from left to right, at concentrations of (0.2, 0.4, 0.6, and 0.8) percent (e–h) with SMM, respectively, used in microencapsulation (40×) (*middle* row). Scan electron microscopy of microencapsulated *Lactobacillus rhamnosus* (MLR) from left to right at concentrations of 0.2, 0.4, 0.6, and 0.8 percent (i–l) with *S. macrosiphon* mucilage (SMM), respectively (bottom row).

The whole beads were produced, containing various concentrations of SMM solutions (Figure 1). The aspect ratio was 1.21 for the beads containing 0.8% SMM. The encapsulation efficiency percentage (EE%) of LR with different SMM percentages is shown in Table 1. The result showed that with the increase in SMM concentration, the EE% increased significantly. The highest EE% was related to 0.8% SMM (97.98%) and was very favorable. One potential explanation for this phenomenon may be related to the combination of alginate with arabinogalactan, which resulted in reduced porosity of the bead surface and prevented bacteria from entering the surrounding environment (Pourakbar et al., 2023).

**TABLE 1:** Layers dimension and color parameters in microencapsulated *Lactobacillus rhamnosus.*

Bead		SMM%			
parameters		0.2	0.4	0.6	0.8
Layers dimension (μm)	Alginate Layer	3268.0 ± 98.90 a	3245.23 ± 89.67 a	3283.52 ± 84.49a	3310.76 ± 90.22a
	SMM Layer	33.91 ± 3.77d	51.48 ± 5.48b	40.92 ± 6.05c	67.92 ± 4.03a
Color parameters	L*	68.11 ± 8.71a	64.22 ± 4.66b	63.00 ± 6.42ab	61.22 ± 5.47b
	a*	−1.89 ± 0.78a	−3.44 ± 0.88a	−3.64 ± 1.22a	−4.11 ± 0.93a
	b*	15.00 ± 2.96c	21.22 ± 2.99b	27.00 ± 5.50a	28.44 ± 4.25a
EE%		84.89 ± 6.17b	91.70 ± 0.98a	94.96 ± 2.48a	97.98 ± 0.78a

*Note*: Data (mean ± standard deviation) are from three replications (*n* = 3). Means followed by different lowercase letters in row had a significant difference (*p* ≤ .05) by the Duncan test.

Abbreviations: EE, encapsulation efficiency; SMM, *Salvia macrosiphon* mucilage.

No significant difference in alginate layer diameter was observed in the produced beads. The study found that varying concentrations of *Balangu* (*Lallemantia royleana*) seed mucilage as a second layer did not result in any significant changes in the thickness of the first layer of sodium alginate in the encapsulation of *Lactobacillus acidophilus* (Sekhavatizadeh & Yaghoobpour, 2023). However, increasing the percentage of SMM concentration caused an increase in the diameter of the SMM layer. Therefore, the 0.8% SMM‐bead had the largest outer layer diameter. In a similar study, Ganje et al. (2024) found that the second layer dimension was increased by tomato seed mucilage concentration. The a* parameter was constant in all samples, and the b* increased significantly with the increase in SMM concentration. The L* decreased significantly with an increase in SMM concentration. Therefore, with the increase in the concentration of SMM, the brightness of the bead decreases and the bead becomes more yellow. Color could be influenced by the type and structure of the bead's second layer. The SMM had a yellow color; therefore, the increase in SMM may lead to yellowness (Salehi & Kashaninejad, 2015).

### 3.2 SEM

The shape of the beads showed the regular morphology of LR in the beads (Figure 1i–l). The images showed that with the increase in SMM concentration, soft and homogeneous surfaces increased in the beads. In a study conducted by Pourjafar et al. (2020), a comparable morphology was observed in *L. acidophilus* and LR encapsulated with chitosan and Eu S100 as wall materials. *L. acidophilus* and LR were encapsulated with chitosan and Eu S100 as wall materials. These results are similar to the results of our study. The wall material structure slightly improves bacterial resistance to adverse conditions (Pourjafar et al., 2020).

### 3.3 Heat tolerance

**FIGURE 2:**
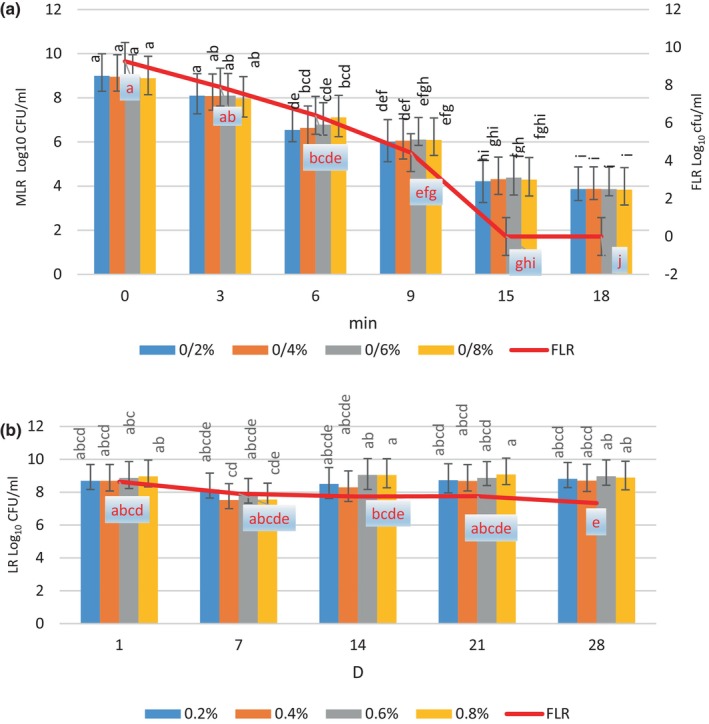
The free *Lactobacillus rhamnosus* (FLR) and microencapsulated *Lactobacillus rhamnosus* (MLR) in different concentration (0.2, 0.4, 0.6, and 0.8%) of *Salvia macrosiphon* mucilage (SMM) survive at 72°C (a) and during storage at 4°C (b). The data (mean ± standard deviation) are from three replications. Means with different lowercase letters had a significant difference (*p* ≤ .05) by the Duncan test.

The data related to the evaluation of the thermal resistance of MLR and FLR at 72°C are presented in Figure 2a. Results showed that the number of FLR and MLR decreased from 9.26 log CFU/mL to 0, and 8.99 to 3.86 in 18 min, respectively. The MLR maintained a minimal probiotic concentration that was more durable than that of free bacteria at 72°C. In 2023, Akbari et al. (2023) conducted a study on the thermal stability of free and microencapsulated *Limosilactobacillus reuteri* at 70°C, and the results showed that cruciferin/alginate capsules were fabricated to encapsulate probiotics, which is consistent with the findings of this research (Akbari et al., 2023). The findings of the current investigation indicated that the survival of MLR was more significant than FLR at 72°C for 15 min. In this regard, Sekhavatizadeh et al. (2024) reported that the survival rate of microencapsulated *Lactobacillus curvatus* encapsulated in *Plantago major* mucilage was 50.15%, and it was greater than the free form (Sekhavatizadeh et al., 2024).

### 3.4 Survival ability of MLR and FLR at 4°C

The survival of FLR and MLR in the MRS medium at 4°C is shown in Figure 2b. The number of FLR decreased from 8.6 log CFU/mL to 7.3 log CFU/mL during storage time. However, the MLR count was reduced from 8.9 log CFU/mL to 8.68 log CFU/mL over the same time. The FLR had the minimum limited probiotic number (10^6^ CFU/mL) during the 21 days of storage, while this time was expanded for MLR. These findings were related to the synergistic effect of SMM and alginate as double‐walled materials in MLR, which leads to more survival ability than FLR during storage time. The MLR count was relatively stable during the storage period. Previous studies have shown that the probiotic viable number in bead samples was more significant than the free probiotic. In the research of Ni et al. (2023), the cell survival of *Lactobacillus plantarum* encapsulated in alginate–gelatine hydrogel beads was diminished to less than 2.1 log CFU/mL. Following 6 days of being stored at 4°C, free *Lactobacillus plantarum* exhibited more viable number reduction than the others. (1.51 log CFU/mL). This result was in contrast with the result of this study. A possible explanation could be associated with the storage medium and time of sampling. The storage period observed in their investigation was shorter than in the current study. Moreover, in our research, we utilized MRS broth as the storage medium, whereas normal saline was used in their study. Therefore, the shelf life of probiotic bacteria can be prolonged by selecting a proper culture media during storage (Ni et al., 2023). The shelf life of probiotics can be prolonged by the selection of proper culture media during storage.

### 3.5 Titratable acidity and pH

**FIGURE 3:**
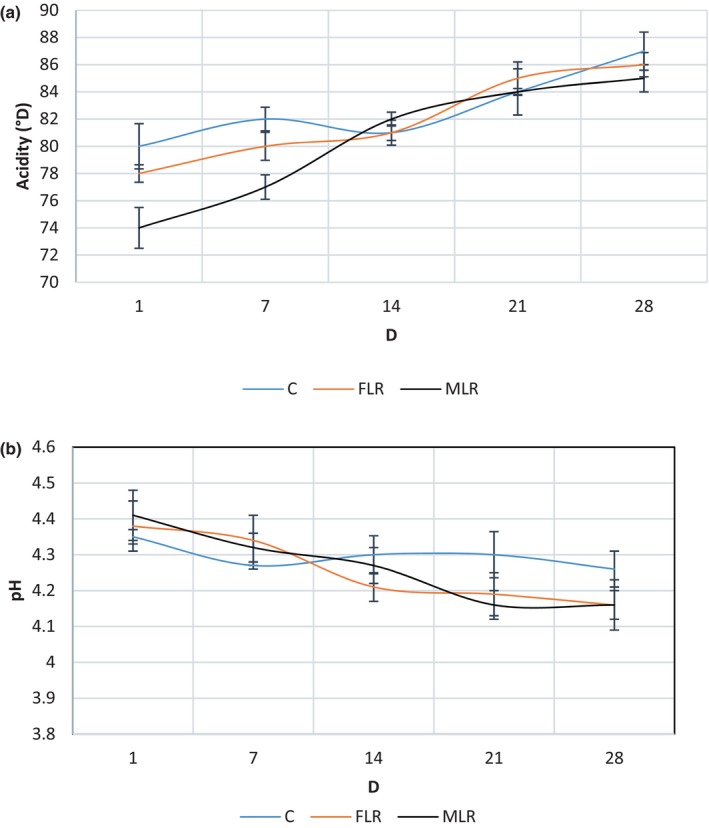
Acidity (a) and pH (b) of free *Lactobacillus rhamnosus* (FLR), microencapsulated *Lactobacillus rhamnosus* (MLR) and control C date yogurt during storage time.

The pH and acidity changes in DY during storage (4°C) and among the supplemented samples are shown in Figure 3a,b. The pH reduction levels in the control sample (C), FLR, and MLR were 0.1, 0.2, and 0.25, respectively. The reduction in pH was greater in FLR and MLR compared to C. *Lactobacillus rhamnosus* is an acid producer, so it decreased pH in FLR and MLR (Dokoohaki et al., 2019). Besides, the production of lactic acid from lactose during storage (post‐acidification) due to the activity of lactic acid bacteria, carbohydrate hydrolysis, and lipid oxidation in the lactic acid accumulation, which finally reduced the pH values of the supplemented samples. Similar results were reported by Pourakbar et al. (2023).

During storage time, the increase in acidity in MLR was the highest among the samples. The external placement of the SMM outside the alginate layer offered two‐fold protection and effectively facilitated the regulation of LR release in the carrier products. It is suggested that the increased acidity and reduced pH may be related to the viable number of LR that existed at the end of the storage time. Enzyme release from probiotic cells that hydrolyzes the sugar components was another factor contributing to increased acidity during storage (Zhu et al., 2020). During the storage time, the pH of the juice decreased while the acidity increased because acidity has an inverse relationship with pH (Ali et al., 2023).

### 3.6 Storage stability

**FIGURE 4:**
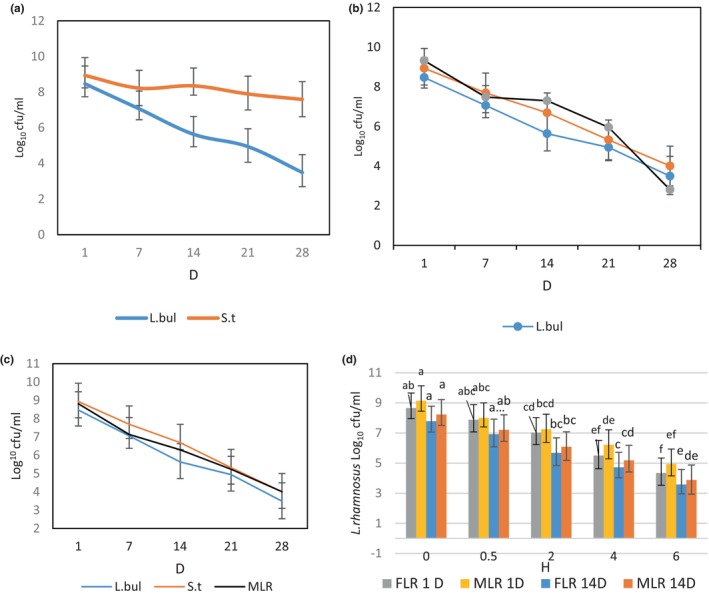
The survival of *Lactobacillus bulgaricus* (*L.blu), Streptococcus thermophilus* (S.t), and *Lactobacillus rhamnosus* (LR) in control‐date yogurt (DY) (C‐DY) (a), DY contains free *Lactobacillus rhamnosus* (FLR‐DY) (b), and DY contains microencapsulated *Lactobacillus rhamnosus* (MLR‐DY) (c) during storage time. The survival of FLR and MLR in date yogurt during simulated gastrointestinal conditions at 1st and 14th days (d) of storage. Data (mean ± standard deviation) are from three replications. Lowercase letters (a–f) show significant differences (*p* ≤ .05) among samples in each parameter during storage time.

Probiotic bacteria are living cells that are sensitive to harsh conditions during food preparation and food storage. Using probiotics in microcapsules can protect them against harsh conditions (Kathiriya et al., 2023; Soltani Lak et al., 2021). In all samples, starter culture bacteria containing *Streptococcus thermophilus* and *Lactobacillus bulgaricus* decreased during the storage time (Figure 4a–c). The viable number of MLR (4.01 log CFU/g) was greater than FLR (2.8 log CFU/g) in supplemented DY samples at the end of storage. The survival rates were 30.0% and 56% for FLR and MLR in the 28th day of storage, respectively. This result agrees with a previous report describing that low temperatures close to above 0°C reduced the rate of detrimental chemical reactions, which could lead to cell damage (Xu et al., 2016). Thus, improving the stability of the beads during storage could reduce the loss of the cells to the medium and positively affect viability (Brinques & Ayub, 2011). Similarly, Kaewiad and Kaewnopparat (2023) used pectin beads containing soybean powder to protect *L. fermentum* as bead wall materials in the extrusion encapsulation technique. The data showed that the survival rate of the encapsulated form was 68.23%. No viable cells from the beads without cryoprotectant could be detected after 2 months of storage (Kaewiad & Kaewnopparat, 2023). The findings are not in line with the current study results. One of the main reasons is probably the different types of wall materials and probiotic strains used.

### 3.7 Survival in SGC

The survival of FLR and MLR under SGC is shown in Figure 4d. On the first day of storage, the survival rates of MLR and FLR were 53% and 50%, respectively. However, by the 14th day of storage, these rates had decreased to 47% for MLR and 45% for FLR. The results demonstrated the protective ability of SMM by acting as a physical barrier. Alginate beads exhibited a porous structure. The biopolymer network (alginate – SMM) could potentially slow the movement of GI fluid across the wall material of beads, resulting in a decreased release of the probiotics from the bead into the GI environment (Parsana et al., 2023).

These findings are consistent with Ali et al. (2023), who utilized alginate with xanthan gum to encapsulate *L. casei*. They provided a better survival ratio for *L. casei* than free ones following 6 h exposure to a bile solution (Ali et al., 2023).

### 3.8 Sensory evaluation

**FIGURE 5:**
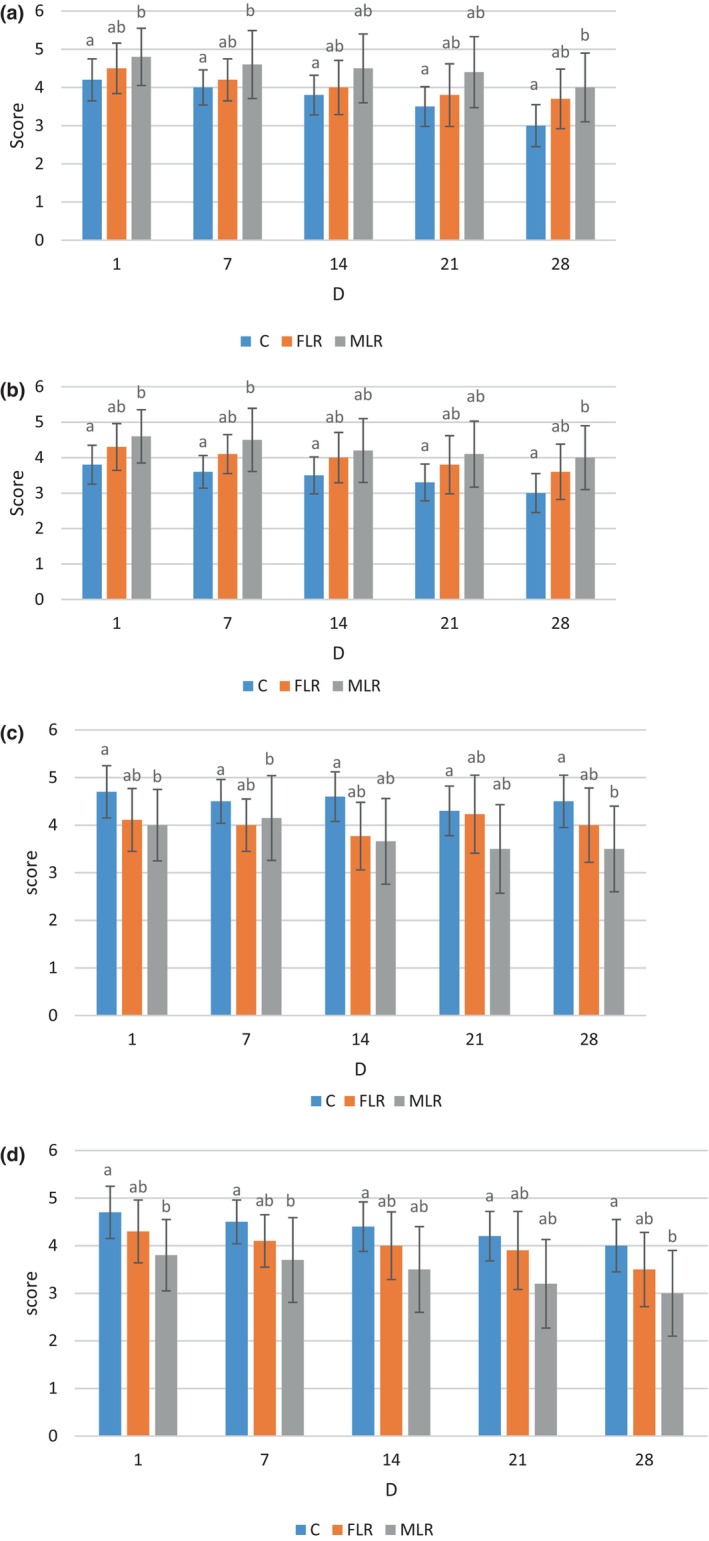
Sensory properties of control (c); microencapsulated *Lactobacillus rhamnosus* (MLR) and free *Lactobacillus rhamnosus* (FLR) during storage time in date yogurt. Sensory parameters consist of odor (a); consistency (b); flavor (c); and color (d). Data (mean ± standard deviation) are from three replications. Lowercase letters (a‐b) show significant different (*p* ≤ .05) among samples in each parameter during storage time.

The sensory evaluation of DY was assessed and shown in Figure 5. MLR showed higher consistency and odor values, possibly because of the release of bead wall material in DY. In the C sample, the flavor, color, and acceptability scores were found to be the highest. The FLR exhibited higher color and acceptability than MLR, which may be attributed to the development of a grainy texture in MLR. Regarding texture parameters, MLR presented higher values of consistency score among the samples, which may be related to the alginate and SMM used. These biopolymers provide a highly viscous medium that can enhance texture. Most importantly, the extrusion method produces beads (above 1000 μm) with a more negative sensory score response. Dong et al. (2020) found that adding microcapsules to a cupcake would not affect the original cupcake's sensory properties. The results we obtained were not consistent with the results reported earlier because the metabolism of microencapsulated probiotics in the cupcake was very slow, which helped cake sensory properties remain unchanged during storage time. In this research, the metabolism of microencapsulated probiotics was different from those used in cupcakes (Dong et al., 2020).

### 3.9 Rheological properties

**FIGURE 6:**
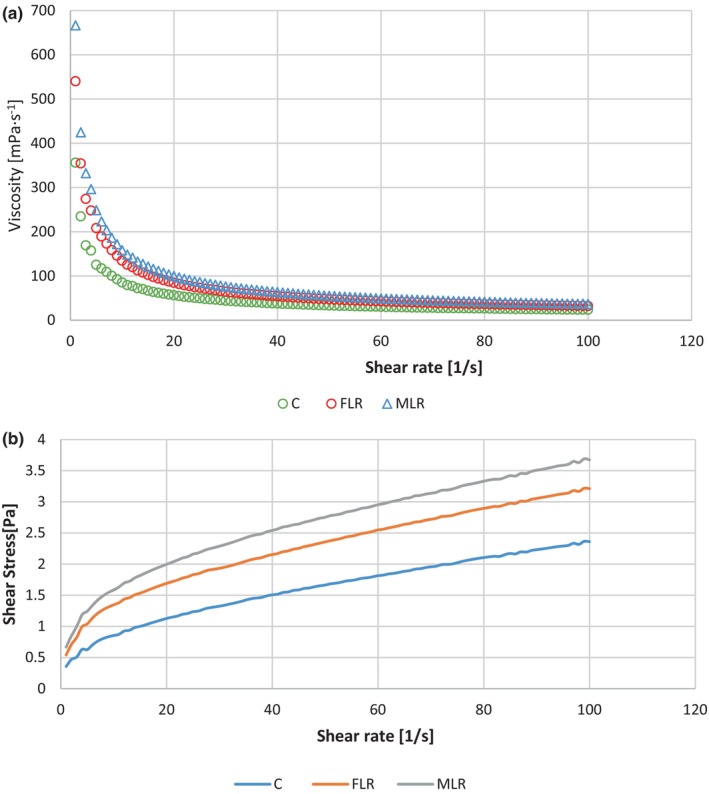
Relationship between viscosity (mPa/s) and shear rate (1/s) (a); shear rate (1/s) shear stress [Pa] (b) in control C; microencapsulated *Lactobacillus rhamnosus* (MLR); and free *Lactobacillus rhamnosus* (FLR) in the 28th day of storage time of date yogurt.

The viscosity of DY in each group decreased simultaneously with the increase in shear rate (Figure 6a), which showed a typical non‐Newtonian behavior (shear thinning). This finding is in line with D'Alessandro et al. (2023). DY presented a pseudo‐plastic fluid. In the initial stages of the flow, pseudo‐plastic fluid exhibited a comparatively high apparent viscosity because of its low shear rate. The ongoing increase in the shear rate led to a gradual decline in the slope of the curve, consequently resulting in a downward trend in the viscosity of the liquid (Torres et al., 2018). This might be attributable to the reduction in particle size due to increasing the shear rate (Sekhavatizadeh et al., 2019). Therefore, the addition of microcapsules may lead to an increase in the apparent viscosity of the DY samples. This outcome was in line with the findings reported by Li et al. (2021). This may result from the interaction between DY protein and SMM (formation of electrostatic complexes). The shear stress exhibited a direct relationship with the increase in shear force (Figure 6b). As expected, all DY samples were characterized by pseudoplastic behavior (Hashim et al., 2021).

### 3.10 SEM image of DY

**FIGURE 7:**
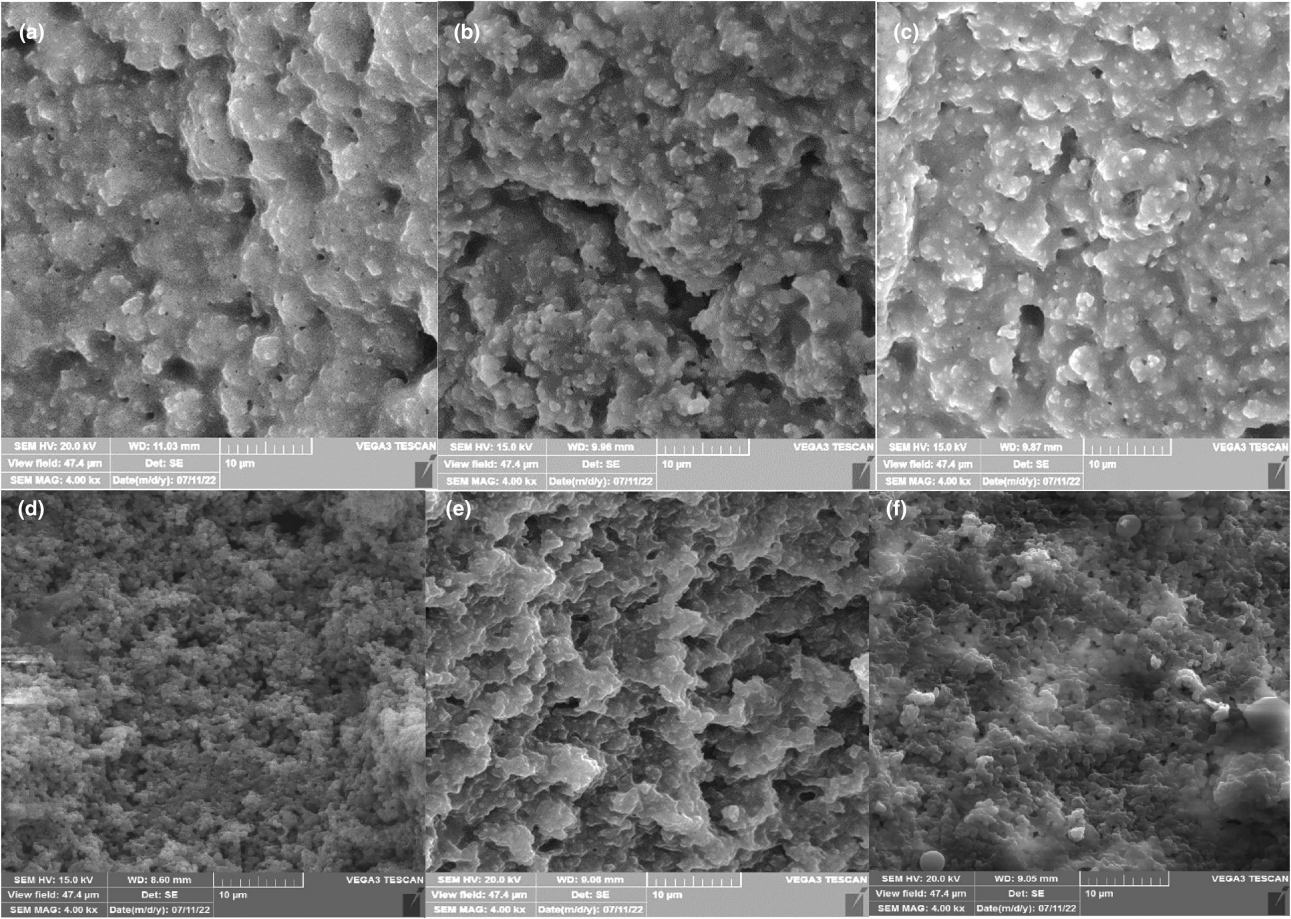
The SEM image of date yogurt contains microencapsulated *Lactobacillus rhamnosus* (MLR), free *Lactobacillus rhamnosus* (FLR), and control C, during storage time. C in 1st (a); FLR in 1st (b); MLR in the 1st (c); C in the 28th (d); FLR in the 28th (e); and MLR in the 28th (f) days of storage period.

The SEM photographs were obtained from the C, MLR, and FLR‐DY samples on the first (Figure 7a–c) and 28th days (Figure 7d–f) of storage (Figure 7). On day 1, the protein network of C‐DY (Figure 7a) was less dense, more open, and contained more cavities than the others. However, the FLR and MLR‐DY samples had a denser structure and less space. Similarly, Pourakbar et al. (2023) reported that on the 1st day of storage, a gel‐like structure was observed in C yogurt, which is thought to be attributed to the protein networks (Pourakbar et al., 2023). It is suggested that the aggregation of casein was more evident in DY samples with non‐encapsulated and encapsulated probiotics. Moreover, the MLR‐DY structure exhibited globular protein aggregates, presumably due to SMM acting as protein binders. The SMM absorbs water and expands and fills the grooves. It will continue to swell during storage. Therefore, the open structure of the protein micelle network was a consequence of some of the solubilizing molecules within the SMM that could enter the DY protein (Sandoval‐Castilla et al., 2004).

## 4 CONCLUSIONS

It was observed that encapsulation of probiotics within SMM and alginate enhanced the cell viability in heat stress, refrigerator conditions, and DY samples during storage. Physicochemical parameters and sensorial characteristics of probiotic DYs were additionally influenced by the MLR. According to the findings, microencapsulation was an effective technique for maintaining the survival ability of LR at the recommended effectiveness level (above 10^6^ CFU/g) in food products. Moreover, it had low adverse effects on the sensorial and physicochemical properties of DY.

## AUTHOR CONTRIBUTIONS

**Seyed Saeed Sekhavatizadeh:** Writing – original draft (lead). **Mahsa Abbasi Saadi:** Investigation (equal). **Hassan Barzegar:** Project administration (equal). **Behrooz Alizadeh Behbahani:** Formal analysis (equal). **Mohammad Amin Mehrnia:** Validation (equal).

## FUNDING INFORMATION

This research did not receive any specific grant from funding agencies in the public, commercial, or not‐for‐profit sectors.

## CONFLICT OF INTEREST STATEMENT

The authors declare that they do not have any conflict of interest.

## ETHICAL REVIEW

This study does not involve any human or animal testing.

## Data Availability

Data will be made available on reasonable request.
